# YAP/TAZ as Molecular Targets in Skeletal Muscle Atrophy and Osteoporosis

**DOI:** 10.14336/AD.2024.0306

**Published:** 2024-03-06

**Authors:** Youngjoo Kwon

**Affiliations:** Department of Food Science and Biotechnology, Ewha Womans University, Seoul, Republic of Korea

**Keywords:** Mechanosensing, mitochondrial function, osteoporosis, skeletal muscle atrophy, YAP/TAZ

## Abstract

Skeletal muscles and bones are closely connected anatomically and functionally. Age-related degeneration in these tissues is associated with physical disability in the elderly and significantly impacts their quality of life. Understanding the mechanisms of age-related musculoskeletal tissue degeneration is crucial for identifying molecular targets for therapeutic interventions for skeletal muscle atrophy and osteoporosis. The Hippo pathway is a recently identified signaling pathway that plays critical roles in development, tissue homeostasis, and regeneration. The Yes-associated protein (YAP) and transcriptional coactivator with PDZ-binding motif (TAZ) are key downstream effectors of the mammalian Hippo signaling pathway. This review highlights the fundamental roles of YAP and TAZ in the homeostatic maintenance and regeneration of skeletal muscles and bones. YAP/TAZ play a significant role in stem cell function by relaying various environmental signals to stem cells. Skeletal muscle atrophy and osteoporosis are related to stem cell dysfunction or senescence triggered by YAP/TAZ dysregulation resulting from reduced mechanosensing and mitochondrial function in stem cells. In contrast, the maintenance of YAP/TAZ activation can suppress stem cell senescence and tissue dysfunction and may be used as a basis for the development of potential therapeutic strategies. Thus, targeting YAP/TAZ holds significant therapeutic potential for alleviating age-related muscle and bone dysfunction and improving the quality of life in the elderly.

## 1. Introduction

Aging is associated with progressive tissue deterioration, which occurs as a result of a continuous decline in the body’s ability to replace cells lost during homeostatic cell turnover and tissue injury [1]. Skeletal muscles and bones are closely connected anatomically and functionally, and age-related defective homeostatic replenishment in these tissues can lead to tissue dysfunctions, such as muscle atrophy and osteoporosis [2, 3]. Moreover, tissue deterioration in skeletal muscles and bones can increase the risk of falls and fractures, making them major contributors to physical disability and decreased quality of life in the elderly [4]. With the increase in life expectancy, the number of elderly individuals with disabilities is expected to rise among the growing elderly population. Thus, it is important to understand the mechanisms underlying age-related musculoskeletal tissue degeneration in order to identify molecular targets for therapeutic intervention to improve physical and functional performance in the elderly.

The Yes-associated protein (YAP) and transcriptional coactivator with PDZ-binding motif (TAZ) are key downstream effectors of the mammalian Hippo pathway. The Hippo pathway is a recently identified signaling pathway that plays critical roles in development, tissue homeostasis, and regeneration [5]. Components of the Hippo signaling pathway were first identified as tumor suppressors in *Drosophila melanogaster* [6]. This evolutionarily conserved pathway is involved in the control of organ size by restraining cell proliferation, promoting apoptosis, and regulating stemness [7]. Since its identification, this pathway has been extensively studied in the field of human cancer. However, subsequent research has revealed its association with other age-related diseases, including neurodegenerative diseases, cardiovascular disease, tissue fibrosis, and tissue atrophy [5, 8]. Notably, reduced YAP/TAZ activity is linked to the decreased regenerative potential in the livers of aged mice [9]. Restoring YAP/TAZ activity by inhibiting the Hippo pathway in aged livers overcomes aging-related defects (*i.e.*, delaying cell cycle re-entry during partial hepatectomy) [9]. Moreover, YAP/TAZ activation enhances osteogenic differentiation and relieves osteoporosis [10, 11]. Concurrently, it inhibits osteoclast formation and bone resorption [11, 12]. YAP/TAZ activity is also important for myogenesis and myoblast proliferation [13-15]. Therefore, the Hippo-YAP/TAZ signaling pathway may be involved in a broad spectrum of pathological conditions, including tissue degeneration. The stimulatory effects of YAP/TAZ activity on the proliferation and differentiation of muscle and skeletal stem cells may serve as a basis for the development of potential therapeutic strategies.

This review first outlines the Hippo-YAP/TAZ pathway and discusses its importance in tissue homeostasis and stem cell function. Moreover, it explores the dysregulation of YAP/TAZ in skeletal muscle atrophy and osteoporosis, with a particular focus on the critical role of YAP/TAZ in responding and integrating various signals from both the intracellular and extracellular environments, and how these signals impact stem cell function. This review aims to advance our understanding of the mechanisms underlying the deterioration of muscle and bone health that accompanies aging and to provide a basis for targeting YAP/TAZ in the prevention and treatment of skeletal muscle atrophy and bone loss.

## 2. Overview of the Hippo-YAP/TAZ pathway

**Figure 1.**
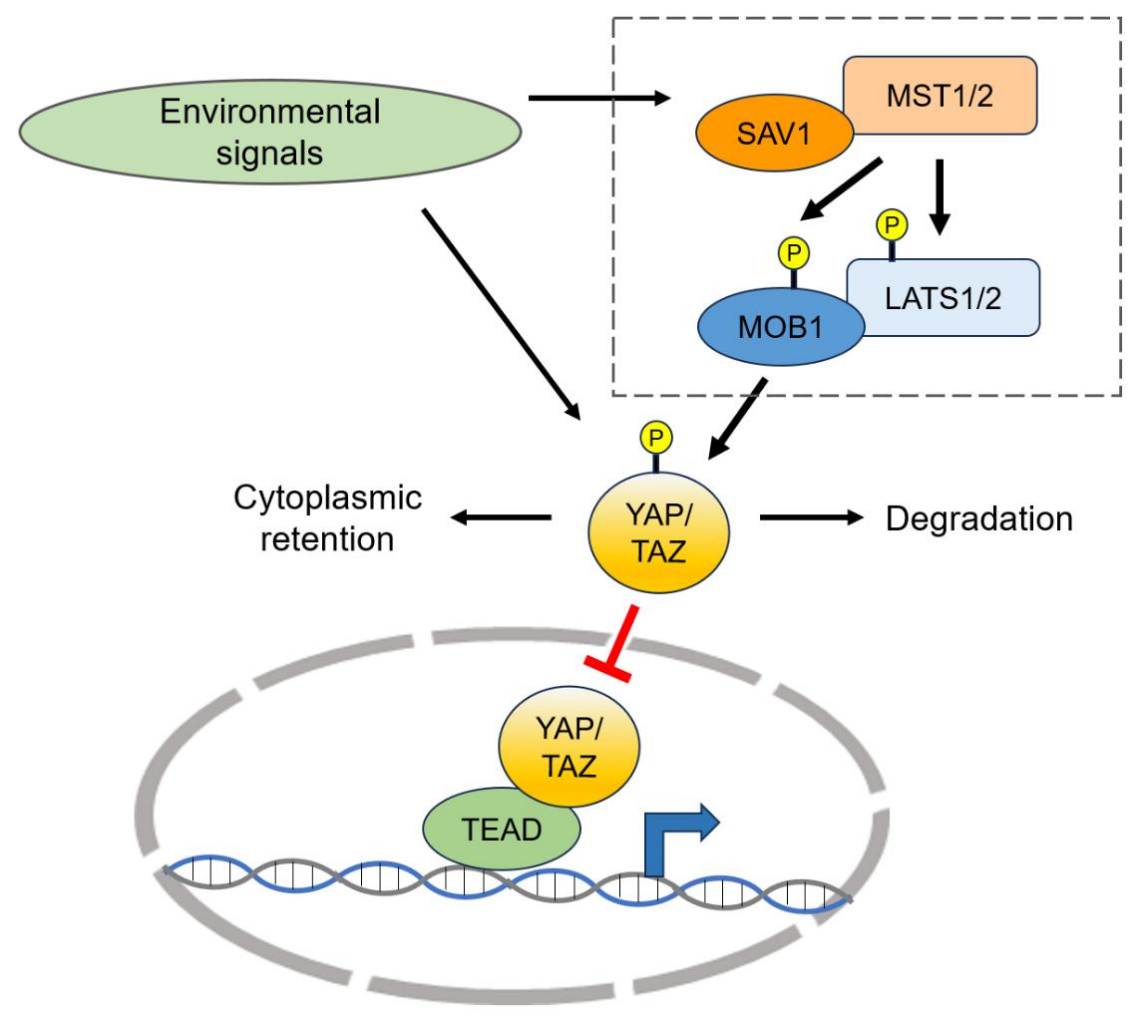
**Core components of the mammalian Hippo-YAP/TAZ pathway and the role of YAP/TAZ as signal integrators**. The mammalian sterile 20-related 1 and 2 kinases (MST1/2) phosphorylate and activate large tumor suppressor kinases 1/2 (LATS1/2). The activation of these Hippo kinases is promoted by the scaffold proteins Salvador homolog 1 (SAV1) and monopolar one binder protein 1 (MOB1), which interact with MST1/2 and LATS1/2, respectively. The activated LATS1/2 can then phosphorylate and inactivate the transcriptional coactivators Yes-associated protein (YAP) and its paralog transcriptional coactivator carrying a PDZ-binding motif (TAZ), leading to their retention in the cytoplasm or degradation, inhibiting their nuclear localization. In the nucleus, YAP and TAZ interact mainly with transcription factors in the transcriptional enhancer factor-domain (TEAD) family to regulate target gene expression. Unlike many other developmental pathways, the Hippo-YAP/TAZ pathway is highly responsive to various cellular signals, controlling cell proliferation, differentiation, and tissue homeostasis. These signals can be relayed through Hippo kinases and are independent of the Hippo kinase in the regulation of YAP/TAZ activation.

After its initial characterization in *Drosophila melanogaster*, the Hippo pathway has been shown to be highly conserved throughout evolution [16]. The Hippo pathway plays a crucial role in controlling organ size and regeneration by regulating cell proliferation, apoptosis, and stemness in *Drosophila* and mammals [17]. At the core of the Hippo signaling pathway is a kinase cascade, where the mammalian sterile 20-related 1 and 2 kinases (MST1/2, mammalian homologs of *Drosophila* Hippo [Hpo]) phosphorylate and activate the large tumor suppressor kinases 1/2 (LATS1/2, mammalian homologs of *Drosophila* Warts [Wts]; Fig. 1). In addition, the activities of MST1/2 are enhanced through their interaction with the Salvador homolog 1 (SAV1) scaffold protein [18]. Another scaffold protein, monopolar one binder protein 1 (MOB1), forms a complex with LATS1/2 and is required for their full activity. MOB1 is phosphorylated by MST1/2, leading conformational changes that promote the interaction between MOB1 and LATS1/2 [19-21]. Upon activation, LATS1/2 phosphorylate and inactivate the transcriptional coactivators YAP and/or its paralog TAZ (mammalian homologs of *Drosophila* Yorkie [Yki]), which are prime mediators of the Hippo signaling pathway (Fig. 1). This activation of the Hippo signaling pathway results in the retention of YAP and TAZ in the cytoplasm or facilitates their degradation, thereby inhibiting their nuclear localization.

As transcriptional coactivators, YAP and TAZ cannot directly bind to DNA. Instead, they translocate into the nucleus and interact with DNA-binding transcription factors to regulate target gene expression following their dephosphorylation. The intracellular localization of YAP/TAZ, predominantly regulated by phosphorylation, is a key determinant of their activity and roles in signal transduction [22]. YAP and TAZ, with an amino acid sequence identity greater than 40%, interact with shared and specific transcriptional partners, exerting overlapping and distinctive functions [23]. The transcription factors in the transcriptional enhancer factor-domain (TEAD) family are key partners of YAP and TAZ. The interaction of these proteins with TEADs in the nucleus triggers the transcription of many genes that control cell proliferation and apoptosis [24]. Moreover, YAP/TAZ interact with other transcription factors important for development and tissue homeostasis, to regulate their transcriptional activity, as described in Section 3.

In contrast with many other developmental pathways that are defined by specific morphogens/hormones and their corresponding receptors, the Hippo-YAP/TAZ pathway stands out for its ability to respond to a wide range of signals. These signals include hormones, mechanical stimuli (e.g., cell-cell or cell-matrix contacts), cellular metabolic status, and various stresses, which play a role in controlling cell proliferation, cell differentiation, and tissue homeostasis [25]. While many of these signals are relayed through Hippo kinases, there have also been reports of Hippo kinase-independent regulation of YAP/TAZ [8]. These observations indicate that YAP and TAZ are regulated by a diverse array of upstream signals and interact with multiple downstream transcription factors involved in tissue homeostasis, highlighting YAP and TAZ as important signal integrators.

## 3. Role of YAP and TAZ in stem cell function and tissue homeostasis

Tissue homeostasis is a process that maintains normal tissue architecture and function. The liver has a unique regenerative ability, allowing it to fully recover its mass and function after injuries, while tightly regulating its size [26]. An growing body of evidence suggests that the Hippo-YAP/TAZ pathway plays an important role in enabling this remarkable regenerative capacity [27]. Notably, the activation of the Hippo signaling pathway undergoes dynamic changes during liver regeneration after partial hepatectomy. For instance, the nuclear YAP level increases whereas the active levels of MST1/2 and LATS1/2 decrease immediately after hepatectomy, returning to baseline levels once the liver size is restored [28]. Deletion of *Yap* and *Taz* in mice prevents efficient cell cycle entry and complete restoration of liver size after partial hepatectomy [27], highlighting the necessity of YAP and TAZ in maintaining homeostasis after liver injury. In contrast to the liver, where tissue replenishment is accomplished by differentiated hepatocytes, many organs contain the terminally differentiated cells that cannot resume proliferation [29]. In these tissues, the maintenance of tissue homeostasis and regeneration dynamics relies on the presence of tissue-specific stem cells, also called adult stem cells [30]. Stem cells have the ability to sustain a stem cell pool throughout their lifespan (self-renewal) and generate various differentiated cell types within the tissue (multipotency) [31]. In addition, tissue-specific stem cells are typically found in a quiescent state within a specific tissue microenvironment, but can transition into a transient amplifying state where they rapidly and extensively proliferate as undifferentiated intermediates before ultimately differentiating into functional tissue cells [32].

In many tissues, tissue-specific stem cells play a key role in the maintenance of tissue homeostasis by replacing missing cells during tissue regeneration and cell turnover [30]. Cell turnover typically involves a continuous cycle of eliminating older cells and replacing them through the division of stem cell progeny. This process serves as an important mechanism to protect organisms from physiological dysfunction, although the rate of cell turnover varies among different organs (from days in the intestinal epithelium to months in the lung epithelium) [33]. Moreover, tissue-specific stem cells initiate appropriate cell division and differentiation in response to cell loss during tissue regeneration [34]. Therefore, in adults, tissue-specific stem cells significantly contribute to maintaining tissue homeostasis. YAP/TAZ may play a crucial role in maintaining the function of tissue-specific stem cells, thereby contributing to tissue homeostasis in various organs beyond the liver, as discussed in Sections 3.1 and 3.2.

### 3.1 Role of YAP/TAZ in stem cell maintenance and expansion

To function properly, stem cells should maintain their stemness, which refers to their ability to proliferate upon activation. YAP may be upregulated in stem cells to help achieve this purpose. In tissues with high cell turnover, such as the skin and intestine, tissue renewal is a continuous process primarily driven by well-characterized stem cell pools [29, 35]. Notably, in these tissues, the spatial distribution of YAP expression is closely linked to proliferative compartments (the base of the crypts or basal layer of the epidermis), where stem and progenitor cells reside [36, 37]. The levels of YAP and TAZ also increase after skin injuries, particularly in the basal cell layer of the epidermis [38].

In comparison, neural stem cells (NSCs) appear early in embryonic development and give rise to diverse types of neurons and glia through neurogenesis [39]. Although most NSCs remain quiescent, with only a small fraction undergoing active proliferation and neurogenesis, they persist throughout adult life [39, 40]. The *YAP* mRNA is also specifically detected in the NSC-containing ventricular zone of the neural tube during chick embryo development [41]. Moreover, its expression is important for the regulation of the transition from quiescence to activation in adult mouse NSCs [40]. Similarly, YAP is highly upregulated upon activation in mouse satellite cells (SCs), which supports the regenerative capability of skeletal muscles, although multiple types of stem cells with muscle-lineage-differentiation potential have been identified [42, 43]. In summary, stem cells express YAP probably upon the activation of proliferation. In contrast, the role of TAZ in stem cell proliferation is less obvious.

YAP may stimulate stem cell proliferation [36, 37, 41]. YAP overexpression increases the number of NSCs partly by inducing cyclin D1 expression and promoting cell cycle progression in the chick neural tube [41]. YAP activation in the mouse intestine through intestinal-specific YAP overexpression also leads to a significant increase in the stem cell compartment [36]. Similarly, overexpression of a mutant YAP in the stem/progenitor cells of the epidermis results in a marked enhancement of stem cell proliferation in the mouse skin [37]. In addition, the constitutive activation of YAP in SCs and myoblasts, which are descendants of activated SCs, results in a significant increase in proliferation, in contrast with the decrease in proliferation observed with YAP knockdown [43]. The activation of SCs into highly proliferative myoblasts depends on the activity of the cAMP response element-binding protein (CREB), which functions through YAP [44]. The MAGUK p55 scaffold protein 7, a target gene of CREB, forms a shuttling complex with angiomotin, facilitating the nuclear transportation of YAP and the maintenance of the proliferative state [44]. Overall, YAP is essential for stem cell proliferation, and its absence results in the failure of regeneration, which is dependent on stem cell proliferation [45, 46].

The YAP-driven proliferation of stem cells may be primarily mediated by TEADs. The number of colony-forming stem cells is reduced in the mouse epidermis when a mutant YAP that cannot interact with TEADs is expressed, despite no change in its expression; this mutant leads to a severely thinned epidermis [47]. In addition, highly proliferative stem/progenitor cells express a TEAD target gene, the glioma-associated oncogene family zinc finger 2 gene [37]. Furthermore, introducing a transcriptionally active mutant of TEAD1 into the chick neural tube increases the number of NCSs, yielding similar results to those seen with YAP overexpression [41]. Thus, TEADs are likely the major transcriptional partners of YAP in promoting stem cell proliferation.

The Hippo pathway primarily controls YAP activity to limit the number of stem cells, but not exclusively [45, 48]. Environmental signals act upstream of the Hippo pathway, or control YAP activity independently of the classical kinases in the Hippo pathway (MST1/2 and LATS1/2). For example, α-catenin, which senses cell density, regulates YAP activity and phosphorylation by modulating the interaction between YAP and protein phosphatase 2A (PP2A) [47]. The 14-3-3 protein and α-catenin form a protein complex with phosphorylated YAP, inhibiting YAP activation by limiting PP2A-mediated YAP dephosphorylation [47]. In this study, MST1/2 deletion did not affect YAP phosphorylation, suggesting the involvement of kinases outside the Hippo pathway in YAP phosphorylation. YAP integrates various environmental signals and transmits them to stem cells to determine their transition from quiescence to proliferation. Environmental signals, such as mechanical loads and reactive oxygen species (ROS), potentially originating from dysfunctional mitochondria, are also important in the regulation of YAP/TAZ-mediated myogenesis and osteogenesis, as elaborated in Sections 4.2 and 4.3.

### 3.2 Role of YAP/TAZ in myogenesis and osteogenesis

The cells that are generated from tissue-specific stem cells through proliferation transition into more specialized cell types through a process called differentiation [49]. This transition from proliferation to differentiation is believed to be largely regulated by the cellular location of YAP [37, 47]. However, studies have also reported that YAP and TAZ play positive regulatory roles in osteogenesis and myogenesis. These conflicting findings can be attributed in part to the use of different sources of stem cells. For example, mesenchymal stem cells (MSCs) have the ability to differentiate into various cell types, such as osteoblasts, chondrocytes, myocytes, and adipocytes, depending on the source (*e.g.*, blood, bone marrow, or fat) and purity of the cells [50]. In addition, skeletal stem cells (SSCs) are a subset of bone marrow stromal cells that are distinct from MSCs. However, they have been used interchangeably because of the ability of MSCs to differentiate into various cell types [51, 52]. Moreover, *in vitro* cell culture models can vary in terms of cell-cell interactions and cellular microenvironments, which may contribute to inconsistent results. In contrast, *in vivo* studies have consistently shown positive regulatory roles for YAP and TAZ in differentiation [53].

In addition, YAP/TAZ can interact with a variety of proteins and crosstalk with various signaling pathways, and their function can be affected by the proteins they interact with [25]. For example, nonreceptor tyrosine kinases (*e.g.*, Src and Yes) can phosphorylate YAP and promote an interaction with the runt domain transcription factor 2 (Runx2), which subsequently targets a subnuclear location to suppress Runx2-mediated osteocalcin promoter activation in rat osteosarcoma cells [54]. In contrast, formation of different protein complexes between YAP and another transcription factor, the activator of protein 2a, enhances osteogenic differentiation in stem cells derived from the apical papilla (dental-tissue-derived MSCs) by inhibiting the formation of the YAP/Runx2 complex [55]. In another case, zinc finger transcription factors Snail and Slug bind to YAP/TAZ, and this YAP/TAZ-Snail/Slug complex cooperatively regulates bone-marrow-derived (BM)-MSC proliferation or differentiation based on the transcription factors they bind [56]. When recruited by TEAD, BM-MSC proliferation is promoted, whereas recruitment by Runx2 leads to osteogenic differentiation [56]. Therefore, the regulation and function of YAP/TAZ are intricate and dependent on the specific context.

In myogenesis and osteogenesis, TAZ is more often associated with the promotion of differentiation. TAZ promotes SC proliferation and enhances myoblast differentiation and maturation into myotubes during the later stages of myogenesis [57]. Moreover, TAZ has been found to physically interact with myoblast determination protein 1 (MyoD) and activate MyoD-dependent gene transcription, thereby enhancing C2C12 myoblast differentiation [15]. In the same study, TAZ-MyoD-dependent myogenin expression was also increased during active muscle differentiation after a freeze injury in mice. Furthermore, TAZ facilitates osteoblast differentiation. Osteoblast-specific overexpression of TAZ increases the bone mineral apposition rate and the number of osteoblasts in mice, thereby enhancing bone formation [58]. Moreover, calvarial cells isolated from TAZ-overexpressing mice show increased osteogenic differentiation but suppressed adipogenic differentiation [58]. TAZ modulates cell-lineage fate during calvarial differentiation by changing the transcription activity of interacting transcription factors; TAZ enhances Runx2-mediated transcriptional activity while simultaneously repressing gene transcription by peroxisome proliferator-activated receptor gamma (PPARγ), which directs BM-derived MSCs into the adipocyte lineage [58]. Similarly, overexpression or pharmacological activation of TAZ enhances the osteogenic differentiation of adipose-derived stem cells (ASCs) and bone formation probably by recruiting the TAZ-Runx2 complex to the promoter of osteocalcin and facilitating its transcription [59]. Conversely, TAZ knockout impairs osteogenic differentiation but promotes adipogenic differentiation of human ASCs [59]. TAZ also regulates receptor activator of nuclear factor κB ligand (RANKL)-induced osteoclast differentiation; global or osteoclast-specific knockout of TAZ causes an increase in osteoclast formation and a decrease in bone mass in mice [12].

With a few exceptions [43, 57, 60], YAP is generally required for myogenesis and osteogenesis [25]. During C2C12 myogenic differentiation, YAP accumulates in the nucleus, while all phosphorylated forms of MST1/2, LATS1, and YAP are downregulated [14]. Moreover, knocking down YAP inhibits C2C12 myoblast differentiation, leading to a decrease in the number, average length, and diameter of myotubes [14]. Conversely, the overexpression of YAP in C2C12 cells increases the activation of extracellular signal-regulated kinase 5 and mitogen-activated protein kinase kinase 5, promoting differentiation into myotubes with increased expression of the myogenin transcription factor and myosin heavy chain [13]. Activation of YAP through MST1/2 inhibition also increases myotube width, myoblast fusion, and myogenin expression in chick muscle cells, ultimately resulting in the formation of fully stratified myofibers with increased size [61]. In terms of osteogenesis, the deleting *Yap* and *Taz* reduces the number of osteoblasts and mineral apposition rates in mice, as well as decreases osteogenic gene induction in isolated osteoprogenitors [11]. YAP is also necessary for the maintenance of bone mass by promoting osteoblast proliferation and differentiation and by suppressing MSC adipogenic potential [62]. This necessity is likely related to the ability of YAP to interact with β-catenin to maintain its nuclear level and promote signaling [62].

In summary, YAP/TAZ are required during osteogenic and myogenic differentiation, as well as stem cell activation and proliferation. However, differentiation involves a gradual progression of signaling changes that result in the sequential transcriptional activation and repression of genes that support progressive development into a specialized cell phenotype [63]. Therefore, the roles of YAP/TAZ in differentiation may be context dependent and complicated by crosstalk with other differentiation regulatory transcription factors [25].

## 4. YAP/TAZ dysregulation in skeletal muscle atrophy and osteoporosis

Skeletal muscle atrophy and osteoporosis result from defective homeostatic replenishment of cells lost during cell turnover and tissue injury, making them major causes of physical disability [2, 3]. Tissue-specific stem cells are required for tissue homeostasis in adults (Section 3). Thus, the dysfunction of these stem cells may be linked to a gradual decline in the ability to replace cells in aging skeletal muscles and bones. During aging, tissue-specific stem cells may lose their ability to self-renew, resulting in a decrease in the stem cell pool [34]. Moreover, they may become less responsive to mitotic signals, leading to a decreased production of uncommitted stem cells and differentiated cells [64]. They may also lose their ability to differentiate appropriately [1]. In such cases, functional cells are lost, or differentiation is altered towards a specific lineage. Consequently, both the number and function of stem cells decrease with age. YAP/TAZ play a crucial role in the maintenance of stem cell function (Section 3). YAP/TAZ dysregulation may significantly contribute to the stem cell dysfunction observed in aging skeletal muscles and bones. Therefore, targeting YAP/TAZ could potentially serve as a strategy to delay stem cell dysfunction, ultimately improving muscle atrophy and osteoporosis during the aging process.

### 4.1 YAP/TAZ dysregulation and stem cell dysfunction with aging

Cell turnover and regeneration in skeletal muscles in adults rely on the continued production of myoblasts from activated SCs. They are normally mitotically quiescent but can be quickly activated to enter the cell cycle and proliferate in response to injury (*e.g.*, ischemia or muscle damage), which is a critical step for the initiation of muscle regeneration [65]. In contrast to skeletal muscle regeneration, the role of SCs in skeletal muscle hypertrophy (*i.e.*, increase in muscle fiber size) remains controversial [66-68]. However, the number of myonuclei per myofiber increases due to various muscle-hypertrophy-promoting conditions, such as strength training, synergist muscle ablation, and reloading after induced atrophy [69]. Moreover, SCs are the source of myonuclei observed during mechanical-load-induced skeletal muscle hypertrophy [70, 71]. The current prevailing view is that SCs actively contribute to skeletal muscle hypertrophy by secreting factors and coming into physical contact with other cell types in adult muscle, in addition to providing additional nuclei to muscle fibers [72].

Bone is constantly remodeled and regenerated through a balance between osteoblast-mediated bone formation and osteoclast-mediated bone resorption, which helps maintain skeletal integrity [52]. SSCs participate in the regulation of life-long bone turnover by generating osteoblasts and chondrocytes, as well as by influencing the differentiation of osteoclasts in coordination with other cell types within the bone marrow stroma [51, 52]. Thus, tissue-specific stem cells are essential for maintaining tissue function in skeletal muscles and bones. Conversely, age-related tissue dysfunction is often linked to a decline in stem cell function, leading to impaired cell turnover and regeneration capacity in skeletal muscles and bones [1-3].

Tissue-specific stem cells can undergo apoptosis and cellular senescence during aging, contributing to stem cell dysfunction [34]. Cellular senescence is a condition of permanent cell cycle arrest in which cells cannot proliferate, even under proper growth conditions, resulting in a decrease in stem cell pools [73]. Senescent cells not only lose the ability to proliferate, but also exhibit senescence-associated secretory phenotypes (SASPs), regulating the functions of neighboring cells by secreting various cytokines, chemokines, and proteases [74]. This may contribute to the alteration of the ability of stem cells to differentiate into specific cell types [31]. In fact, aging is associated with a shift in lineage differentiation from osteogenesis to adipogenesis, along with a decrease in the MSC pool [75]. Histological analyses of bone biopsies from aged and osteoporotic individuals have revealed an inverse relationship between bone mass and the formation of marrow adipose tissue (MAT) [76]. This reciprocal relationship between high MAT and low bone density is linked to the replicative exhaustion and abnormal differentiation of SSCs, directing SSC commitment toward adipogenic differentiation over osteogenic differentiation [77, 78]. The greater propensity to differentiate toward adipocytes over osteoblasts is associated with a higher risk of bone fracture during osteoporosis and aging [79, 80]. BM-MSCs derived from old mice also exhibit a decreased ability to produce osteoblasts and chondrocytes but generate more stromal lineages expressing high levels of proinflammatory and pro-resorptive cytokines, leading to age-associated bone loss [2]. Therefore, the senescence of stem cells reduces stem cell pools and alters lineage decision, contributing to defective tissue homeostasis and dysfunction. Preventing stem cell senescence and maintaining stem cell function (activating quiescent stem cells and making lineage decisions) may improve regenerative capability in various tissues, including skeletal muscles and bones.

YAP-TEAD complexes play a role in the activation of quiescent stem cells for proliferation (Section 3.1). Conversely, the activation of Hippo kinases is associated with senescence phenotypes [81, 82]. Notably, the nuclear localization of YAP decreases in aged human dental-pulp-derived MSCs and in cells undergoing replicative senescence in culture [83]. YAP activation using a mutant YAP with a deletion in the S127 phosphorylation site (YAP S127) prevents senescence of human pulp-derived MSCs in a TEAD4-dependent manner and restores their regenerative capabilities upon subcutaneous implantation in immunocompromised mice [83]. Similarly, YAP knockout results in premature senescence, whereas YAP overexpression suppresses the senescence phenotype by cooperating with TEAD to trigger the expression of forkhead box (Fox) D1 in human MSCs [84]. In addition, the regulation of YAP/TAZ activity is important for SSC and MSC specification towards osteogenesis or adipogenesis (Section 3.2). Therefore, YAP/TAZ play a vital role in preventing stem cell exhaustion and biased differentiation by suppressing stem cell senescence and maintaining stem cell function. Furthermore, YAP/TAZ dysregulation that causes stem cell dysfunction can occur not only in stem cells, but also in cellular components of the stem cell niche, which serves as a unique microenvironment to support the self-renewal and multipotential activities of stem cells (Section 4.2). At the stem cell level, YAP/TAZ dysregulation can be caused by changes in the extracellular microenvironment (e.g., extracellular matrices) as well as in the intracellular environment (e.g., ROS levels) that occur during aging (Section 4.3).

### 4.2. YAP/TAZ dysregulation in mediating stem cell dysfunction induced by decreased mechanosensing during aging

Cells perceive the physical, mechanical, and architectural properties of their microenvironment by sensing various physical forces, including shear, tensile, and compression impulses, which can be generated by substrate stiffness, cell contractility, and forces produced by adjacent cells [85]. Mechanotransduction is characterized by cellular processes through which mechanical impulses are converted into biochemical signals that stimulate cellular processes, such as transcriptional regulation and signaling pathway activation, thereby eliciting cellular responses [86]. In addition to soluble signals (*e.g.*, signaling ligands), mechanical cues are essential in the regulation of various cellular events, including cell survival, migration, proliferation, and differentiation [86]. YAP/TAZ are widely recognized as key mechanosensory and mechanotransducer entities [87]. The mechanical stimuli induced by matrix stiffness, cell spreading, and fluid shear stress regulate YAP/TAZ activity through complex interactions between focal adhesion, interconnected actin fibers, Ras homolog family member A (RhoA), various components of the Hippo signaling pathway, and other cellular signaling networks (*e.g.*, Wnt/β-catenin) [88-90].

Senescence is a common feature in late-passage fibroblast cultures. Notably, a decrease in the mechanosensing ability of fibroblasts is associated with senescence phenotypes through YAP/TAZ inactivation [91]. In dermal fibroblasts, enhancing endogenous mechanotransduction (YAP/TAZ activation) by using an integrin agonist suppresses SASP, while senescence is induced by reducing RhoA activity or YAP/TAZ inactivation [92]. Moreover, inducing senescence in late-passage fibroblasts is functionally related to decreased YAP/TAZ activity, and YAP/TAZ inactivation causes a remarkable accumulation of senescent cells [92]. Therefore, YAP/TAZ dysregulation is connected to an impaired mechanosensing ability and the resulting senescence phenotypes in stem cells and fibroblasts.

The muscle is highly responsive to mechanical loading during the regulation of muscle growth; mechanical loading induces muscle hypertrophy, while disuse leads to muscle atrophy [93]. Similarly, mechanical loading plays a crucial role in bone formation and the maintenance of bone mass, whereas unloading can result in bone loss and fractures [94, 95]. Therefore, the function of YAP/TAZ as sensors and transducers of mechanical signals suggests their involvement in the growth and maintenance of muscle and bone mass [57]. In other words, mechanical cues control YAP/TAZ activity to regulate the maintenance of muscle and bone mass.

The importance of YAP/TAZ in maintaining muscle mass was demonstrated in a previous study, where YAP expression levels were manipulated through intramuscular delivery of a short-hairpin RNA targeting mouse *Yap* or a vector DNA carrying *Yap* into a mouse limb muscle [68]. The study found that YAP knockout led to a decrease in muscle mass and the cross-sectional area (CSA) of myofibers, while YAP overexpression promoted skeletal muscle fiber hypertrophy in a TEAD-dependent manner. Therefore, the study indicates the importance of YAP expression in maintaining basal skeletal muscle mass. Moreover, YAP expression increases in response to mechanical overload induced by synergist ablation surgery in mice, with YAP overexpression being sufficient to induce muscle fiber hypertrophy [96]. However, previous studies have focused exclusively on the role of YAP in protein synthesis. Muscle hypertrophy is accompanied not only by an increase in protein synthesis, but also by an increase in the number of myonuclei, which depends on SC function [97, 98] (Section 4.1). Therefore, as a mechanosensor, YAP may also affect SC function in mediating muscle hypertrophy induced by mechanical overload. Moreover, mechanical cues play a crucial role in stem cell lineage specification [88, 99, 100]. Mechanical signals can be transmitted from the extracellular matrix (ECM) in contact with stem cells and stromal cells adjacent to them, facilitating mechanotransduction in stem cells (Sections 4.2.1 & 4.2.2; Table 1).

**Table 1:** Effect of YAP/TAZ activation in response to mechanical stimuli on stem cell function.

Sites of YAP/TAZ activation	Mediator (or source) of mechanical stimuli	Outcomes in stem cells	Reference
**Stomal cells**	Fibro/adipogenic progenitors • Production of Thrombospondin-1	Muscle stem cell • Proliferation	[101]
	Surrounding mesenchymal cells • Expression of the bone morphogenetic protein (BMP)	Zebrafish osteoblast • Differentiation • BMP signaling	[102]
	MLO-Y4 osteocytes • Expression of chemokines	-	[103]
	MLO-Y4 osteocytes • Increase in Wnt expression	Osteoblast • Increase in bone formation	[104]
	Mouse osteocytes • Expression of matrix-remodeling enzymes for bone remodeling	Osteoblast/osteoclast • Bone remodeling required for bone strength	[105]
**Stem cells**	Extracellular matrix (ECM) stiffness	Mesenchymal stem cells (MSCs) • Inhibition of adipogenesis • Promotion of osteogenesis	[88]
	Hydrogel stiffness	C2C12 myoblasts • Formation of myotubes with a cylindrical morphology	[106]
	ECM stiffness	ST2 mouse MSCs • Osteogenic lineage commitment	[107]

#### 4.2.1. YAP/TAZ activity in stromal cells adjacent to stem cells during mechanosensing

The impairment of mechanical load sensing may be one of the age-related SC dysfunctions [108]. Aged SCs are less responsive to shear strength force [109]. Intriguingly, YAP/TAZ activity and the number of cells responsive to actomyosin tension decrease remarkably during physiological aging [92]. Moreover, YAP/TAZ activity is primarily found in stromal cells, suggesting a progressive decrease in YAP/TAZ mechanosignaling in stromal cells with aging [92]. In the same study, the conditional knockout of YAP/TAZ in stromal cells exacerbated aging phenotypes, while increasing YAP activity slowed down aging traits resulting from physiological aging or induced by compliant ECM (decrease in mechanical forces). Moreover, mechanotransduction through YAP/TAZ activation suppressed cyclic GMP-AMP synthase-stimulator of interferon gene signaling, a proinflammatory pathway responding to cytoplasmic DNA accumulation [92]. Therefore, the presence of mechanotransduction in stromal cells is critical for preventing aging phenotypes, including the suppression of stem cell senescence.

**Figure 2.**
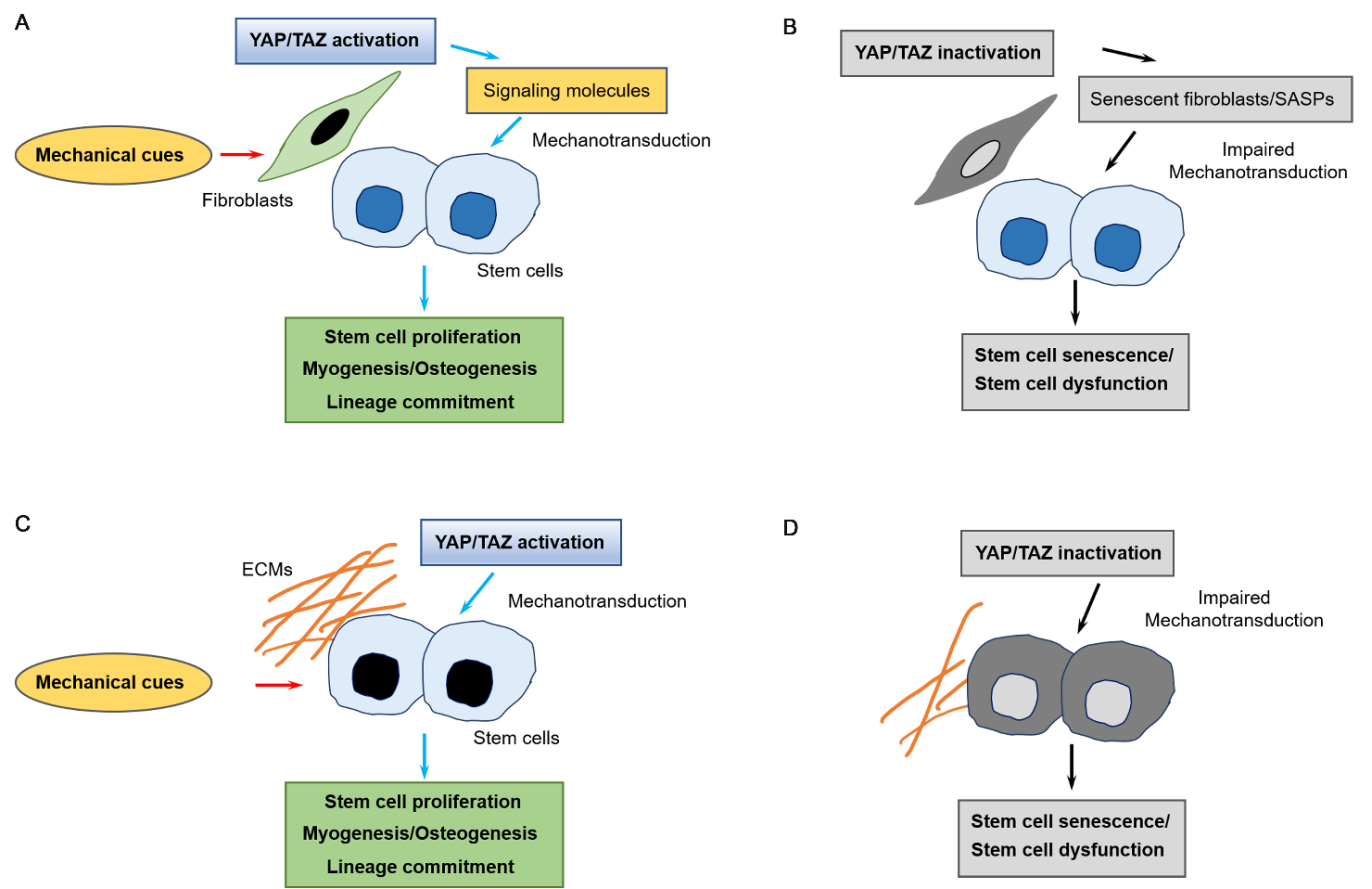
**Role of YAP/TAZ in mechanosensing induced by stromal cells adjacent to stem cells and the ECM, which contacts stem cells**. Mechanical cues can be sensed by stromal cells adjacent to stem cells, which may induce the activation of YAP/TAZ in stromal cells and the secretion of molecules that act on stem cells, promoting their proliferation and differentiation (**A**). However, aging alters extracellular matrix organization, which leads to reduced YAP/TAZ activation in stromal cells adjacent to stem cells, thereby inducing senescence and senescence-associated secretory phenotypes (SASPs). Consequently, mechanotransduction is reduced, and senescence is induced in stem cells (**B**). Alternatively, stem cells sense mechanical cues and induce YAP/TAZ activation, resulting in the upregulation of genes associated with stem cell proliferation and differentiation (**C**). During aging, a disorganized ECM affects YAP/TAZ activity in stem cells, leading to reduced mechanotransduction and stem cell senescence (**D**).

Coincidentally, the initiation of SC proliferation in overloaded muscles depends on YAP/TAZ activity in stromal cells, also known as fibro/adipogenic progenitors [101]. Increased mechanical load induces the nuclear localization of YAP/TAZ in fibro/adipogenic progenitors, resulting in the local production of thrombospondin-1, which in turn drives SC proliferation through CD47 signaling [101]. Thus, SC activation and fate decision rely on the coordinated responses from adjacent myofibers, fibroblasts, and adipocytes in physiological conditions [108]. YAP is also essential for fin regeneration in zebrafish [102, 110]. YAP is localized in the nuclei of stromal cells, activating bone morphogenic protein (BMP) signaling in adjacent osteoblasts, for differentiation during caudal fin regeneration; in contrast, osteoblasts mainly express YAP in the cytoplasm [102]. In addition, the regenerative role of YAP is dependent on mechanical tension during fin regeneration [110]. Previous studies have suggested that YAP/TAZ activity in stromal cells may be crucial for sensing mechanical signals, which are then transmitted to neighboring stem cells for their activation and differentiation (Fig. 2A). The reduced mechanosensing ability observed during aging may cause stem cell senescence by decreasing YAP/TAZ activity in surrounding stromal cells (Fig. 2B).

In mammalian skeletal tissue, osteocytes may function similarly to the fibro/adipogenic progenitors found in muscle tissues. Osteocytes, which originate from mature osteoblasts, are the most abundant cells in bones [111]. Residing within the bone matrix, they sense mechanical forces and transmit bone anabolic signals to osteoblasts and osteoclasts through direct cell-to-cell interactions and the secretion of various molecules [111, 112]. For example, soluble factors produced by MLO-Y4 osteocytes stimulate the proliferation, differentiation, and bone formation capacity (*e.g.*, deposition of calcium) of mouse BM-derived MSCs [113]. Moreover, exosomes released by MLO-Y4 cells exposed to mechanical loads (*e.g.*, cyclic stretch) promote the proliferation and osteogenic differentiation of human periodontal ligament stem cells (PLSCs) [114]. Therefore, osteocytes likely sense mechanical stimuli and regulate stem cell proliferation and osteogenic differentiation by secreting soluble molecules within the bone.

In line with the anticipated osteocytic function of sensing mechanical stimuli, mechanical forces induce YAP/TAZ activation in osteocytes [103-105]. Applying mechanical load to a 3D culture of MLO-Y4 cells increases the nuclear translocation of YAP/TAZ and induces the expression of chemokines [103]. Similarly, in MLO-Y4 cells, the expression of Piezo type mechanosensitive ion channel component 1 *(Piezo1*) is upregulated in response to fluid shear stress, leading to enhanced nuclear translocation of YAP and TAZ, which in turn induces *Wnt* expression [104]. The same study further demonstrated that conditional deletion of *Piezo1* in osteoblasts and osteocytes leads to a failure to increase in bone mineral density (BMD) and *Wnt* expression in response to mechanical loads in mice [104]. Moreover, in a mouse model with selective deletion of YAP/TAZ in osteocytes, bone mass is reduced due to increased osteoclast activity and decreased osteogenesis, suggesting that osteocytes can provide soluble factors that coordinate the activity of osteoblasts and osteoclasts [105]. Collectively, previous research suggests that osteocytes regulate YAP/TAZ activation in response to mechanical loads to trigger the homeostatic activity of osteoblasts and osteoclasts. Therefore, mechanical cues may be sensed by modulating the YAP/TAZ activity in stromal cells (*e.g.*, fibro/adipogenic progenitors, and osteocytes), followed by their transmission to adjacent stem cells for muscle or bone formation. Aging is associated with a decline in mechanosensing and signaling by stromal cells surrounding stem cells, ultimately leading to stem cell dysfunction (Fig. 2A & B).

However, YAP/TAZ mechanosignaling in stromal cells has also been implicated in tissue fibrosis [115, 116]. Nuclear expression of YAP/TAZ is high in affected tissues in human fibrotic diseases (*e.g.*, idiopathic pulmonary fibrosis [IPF] and systemic sclerosis) [117-119] and experimentally-induced fibrotic conditions (*e.g.*, myocardial infarction, diabetes, and chronic colitis) in mice [120-124]. In addition, pharmacological disruption of YAP-TEAD interaction using verteporfin reduces fibrosis in a mouse model of cardiac fibrosis and fibrotic renal injury [116, 121]. These findings support the involvement of YAP/TAZ in fibrosis *in vivo*. Moreover, YAP/TAZ activation in stromal cells, in particular, has been suggested to play central roles in the progression of fibrosis [115, 118, 124, 125]. For example, the transfer of fibroblasts carrying doxycycline-inducible constitutively active mutants of YAP (YAP 5SA) or TAZ (TAZ 4SA) drove fibrosis upon YAP/TAZ activation [118]. Consistently, it was demonstrated that cardiac fibrosis was relieved by fibroblast-specific *Yap*/*Taz* deletion but increased by fibroblast-specific YAP 5SA expression after myocardial infarction in mice [122]. Similarly, fibroblast-specific *Yap*/*Taz* knockout mice exhibited attenuated bleomycin-induced lung fibrosis, unilateral ureteral obstruction-induced kidney fibrosis, and CCl_4_-induced liver fibrosis [115]. Moreover, nuclear localization of YAP/TAZ increased in fibroblasts positive for α-smooth muscle actin, which occurred early after ischemia/reperfusion injury in mouse kidney when fibrosis had just begun to appear [115].

Indeed, the mechanosensing ability of stromal cells through YAP/TAZ activity plays a central role in the fibrotic process, as well as in maintaining the proper function of stem cells. YAP/TAZ nuclear localization in stromal cells is induced by stiff ECM, which mediates fibroblast activation and further ECM synthesis, ultimately leading to tissue fibrosis [116, 118, 121, 123, 124]. Intriguingly, the microenvironment (*e.g.*, ECM stiffness) rather than the origin of cells determines YAP/TAZ activation in stromal cells and the progression of fibrosis. Stiff ECM increases YAP/TAZ nuclear localization in fibroblasts regardless of whether they are derived from normal lung or IPF-affected lung [118]. The same study also highlights the microenvironmental dependence in YAP/TAZ activation-mediated fibrosis by demonstrating a noticeable reverse effect (*e.g.*, decrease in ECM protein accumulation, proliferation, and contractile force generation) of YAP/TAZ knockdown only in fibroblasts growing on stiff but not soft matrices. Therefore, the dynamics of YAP/TAZ activity in stromal cells heavily rely on the mechanical microenvironment. These findings suggested that difference in mechanical microenvironment in various medical conditions (tissue fibrosis *vs.* tissue atrophy) may confer distinct potentials to residing stromal cells (activation *vs.* senescence). Therefore, it is necessary to further understand the spatio-temporal features of YAP/TAZ activation and elucidate the upstream and downstream components of the YAP/TAZ pathway in different pathological contexts in order to specifically target this pathway to enhance stem cell function without promoting fibrosis.

#### 4.2.2. YAP/TAZ activity in stem cells on ECM contacts during mechanosensing

Aging affects cellular mechanical properties and the ability to promptly rearrange the cytoskeleton. Moreover, aging is accompanied by compositional and structural changes in the ECM, which provides structural and mechanical support for tissue integrity, as well as other cellular functions [126]. These changes in cellular and extracellular mechanical properties may lead to alterations in the mechanosensing and mechano-transducing abilities of cells. Studies using controlled mechanical stimulation have demonstrated that physical force can influence lineage-specific differentiation, with YAP/TAZ playing a crucial role in relaying these mechanical signals from the cellular microenvironment [88, 99, 127]. For example, the stiffness of hydrogels is important for the differentiation of C2C12 myoblasts into myotubes with a typical cylindrical morphology; this stiffness-dependent regulation requires the nuclear localization of YAP/TAZ [106]. Furthermore, BM-MSCs can differentiate into osteoblasts or adipocytes based on specific microenvironments or mechanical regulations [88, 99]. The specialization of MSC lineage between osteoblasts and adipocytes relies on substrate stiffness and cell shape [88]. The osteoblast differentiation requires nuclear translocation of YAP and TAZ induced by a stiff substrate and cell spreading [88]. Similarly, ECM stiffness can regulate the commitment of human MSCs and ST2 mouse BM-MSCs to either the adipocyte or osteoblast lineages [107]. A rigid substrate promotes the nuclear localization of TAZ, inhibiting adipocyte differentiation [107]. Vinculin, a cytoskeletal focal adhesion protein, is necessary for the enhanced nuclear localization and activity of TAZ, independent of the Hippo kinases [107]. In another scenario, two distinct pathways, mechanical stimulation and BMP2 signaling, converge at the transcriptional level to induce the osteogenic commitment of C2C12 cells, suggesting the multilevel control of cell differentiation [128]. The transcriptional activation of genes required for osteogenic differentiation depends on the cooperation between BMP2 pathway-associated Smad 1/5/8 heteromeric complexes and YAP/TAZ nuclear translocation regulated by cytoskeletal tension [128]. Therefore, mechanical signals stemming from substrate stiffness or shear stress can control the cell-type-specific differentiation of MSCs, contingent on the proper regulation of YAP/TAZ [85]. The dynamic interactions between stem cells and their niche components are crucial for the regulation of the quiescence, activation, proliferation, and differentiation of stem cells [63]. YAP/TAZ act as mediators between environmental signals and tissue-specific stem cells, transmitting physiological demands to a stem cell response [29, 53]. Aging disrupts mechanical signal transduction due to changes in cell and environmental mechanical properties, contributing to the dysregulation of YAP/TAZ and subsequent stem cell dysfunction (Fig. 2C & D).

### 4.3 YAP/TAZ dysregulation in mediating stem cell dysfunction induced by mitochondrial dysfunction during aging

Aging is associated with mitochondrial dysfunction, as well as reduced mechanosensing ability [129, 130]. Mitochondria are one of the main sources of ROS, which are produced during mitochondrial respiration, a normal metabolic process that converts the energy stored in nutrients into adenosine triphosphate (ATP) [131]. Dysfunction in the mitochondrial respiratory chain and inefficient oxidative phosphorylation may lead to additional electron leakage and an increase in ROS generation. Increased ROS may induce DNA damage and telomere shortening, ultimately contributing to cellular senescence [132]. The disruption of mitochondria, triggered by the deletion of mitochondrial Sirtuins (*e.g.*, Sirt3 and Sirt5) induces senescence [133]. Similarly, the chemical disruption of mitochondria using ethidium bromide or the electron transport chain inhibitor rotenone also causes senescence [133]. Conversely, high ROS levels can induce mitochondrial dysfunction. An oxidative environment decreases the mitochondrial membrane potential and ATP production, inducing mitochondrial dysfunction in MSCs [134]. Moreover, the oxidative stress-induced mitochondrial dysfunction causes MSC senescence, whereas the reduction of ROS levels through the addition of mitoquinone to MSC cultures rescues MSCs from senescence [134]. In conclusion, dysregulated ROS levels and mitochondrial dysfunction are interconnected, contributing to cellular senescence during the aging process.

**Figure 3.**
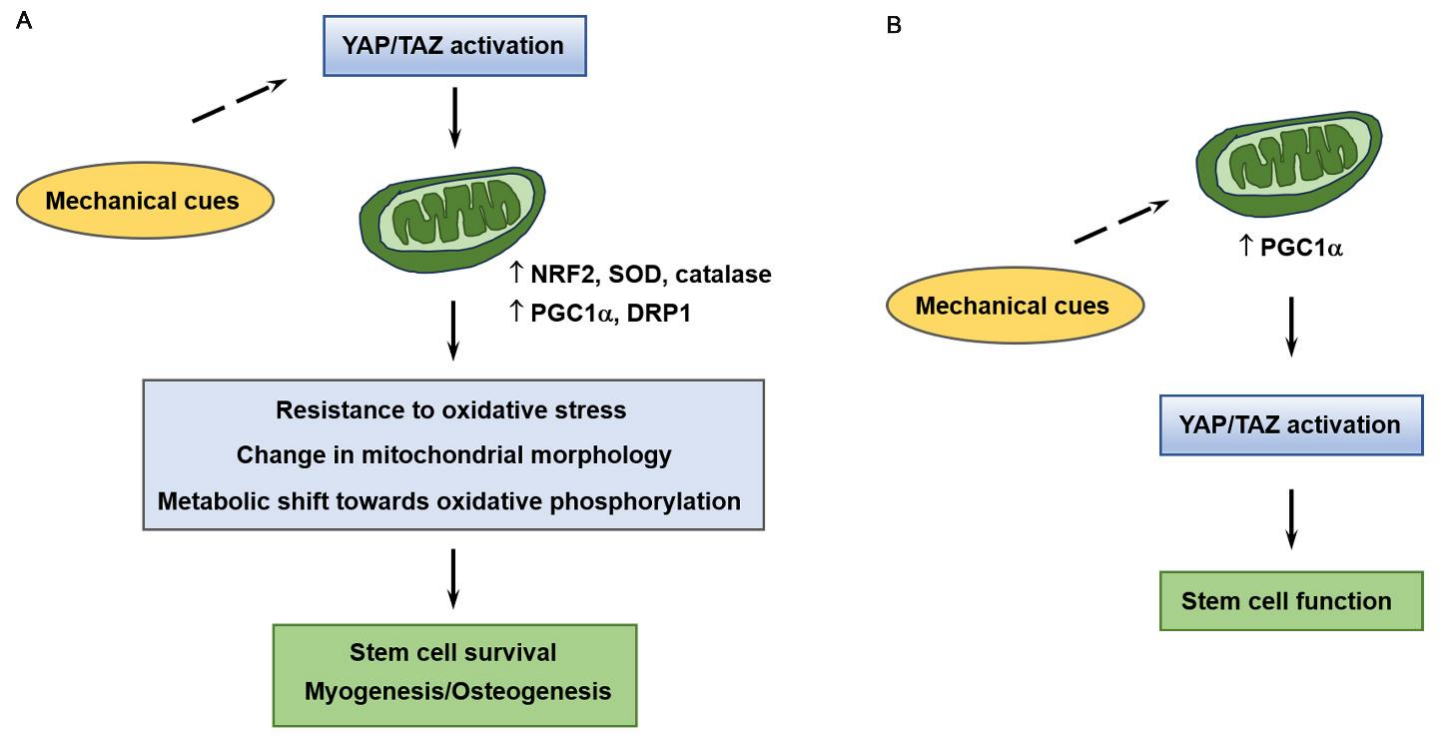
**Role of YAP/TAZ in mitochondrial function**. YAP/TAZ upregulate antioxidant proteins (*e.g.*, nuclear factor erythroid 2-related factor 2 [NRF2], superoxide dismutase [SOD], and catalase) to compensate for the increase in ROS accumulation during stem cell differentiation. YAP/TAZ also upregulate proteins associated with mitochondrial homeostasis, including peroxisome proliferator-activated receptor-gamma coactivator (PGC)-1α and dynamin-related protein 1 (DRP1). Thus, the activation of YAP/TAZ induces the expression of crucial genes related to mitochondrial function, enhancing resistance to oxidative stress, morphological changes, and a metabolic shift towards oxidative phosphorylation (**A**). Conversely, mitochondrial function may influence YAP/TAZ activity and the maintenance of stem cell function (**B**). Because of the involvement of the mechanosensing ability in mitochondrial function, a reduced mechanosensing ability may induce the dysregulation of YAP/TAZ, which causes mitochondrial and stem cell dysfunction. Alternatively, mitochondrial dysfunction resulting from reduced mechanosensing may contribute to YAP/TAZ dysregulation and subsequent stem cell dysfunction.

Mitochondrial metabolism and dynamics play a crucial role in maintaining stem cell homeostasis. Notably, mitochondrial function affects osteogenesis and myogenesis [135, 136], suggesting that dysfunctional mitochondria may contribute to age-related tissue degeneration. Moreover, compelling evidence suggests that mitochondrial dynamics and mechanosensing interact during myogenesis and osteogenesis [137, 138]. Alterations in mechanosensing ability may induce mitochondrial dysfunction. Thus, mitochondrial dysfunction may mediate stem cell dysfunction induced by reduced mechanosensing ability. This indicates that dysregulation of YAP/TAZ may be associated with dysfunctional mitochondria, as YAP/TAZ activity is crucial for mechanotransduction in stem cells for proper function (Section 4.2). Moreover, YAP/TAZ are important for maintaining mitochondrial function [139]. Hence, reduced mechanosensing ability induces the dysregulation of YAP/TAZ, resulting in mitochondrial dysfunction and stem cell dysfunction (Fig. 3A). Alternatively, mitochondrial dysfunction induced by reduced mechanosensing may contribute to the dysregulation of YAP/TAZ, ultimately leading to stem cell dysfunction (Fig. 3B).

#### 4.3.1 Role of mitochondria in myogenesis and osteogenesis

The primary roles of mitochondria are to produce ATP through oxidative phosphorylation and to regulate cellular metabolism [140]. They also perform various fundamental functions in cells, including executing important biosynthetic activities, controlling intracellular Ca^2+^ metabolism and signaling, regulating thermogenesis, generating cellular ROS, and regulating different types of cell death [141, 142]. Thus, mitochondria are crucial in eukaryotic cells, as any disruption in their normal function can be detrimental to cell viability [143]. Consequently, mitochondrial quality is controlled extensively to protect them from stress and damage. This quality control process involves recognizing and correcting issues in the mitochondrial proteome [143]. However, if damage exceeds the capabilities of protein repair systems, a more extensive response is activated, involving the coordinated generation of new mitochondria through mitochondrial biogenesis and the removal of damaged mitochondria through mitophagy [140, 144]. The maintenance of mitochondrial integrity and homeostasis is also ensured through continual fusion and fission, collectively known as mitochondrial dynamics [145]. As a result, mitochondria exhibit a remarkable plasticity, constantly undergoing mitochondrial fission and fusion, biogenesis, and mitophagy to control their morphology, quantity, quality, and turnover.

Mitochondrial remodeling is closely related to mitochondrial function [146]. In response to changes in energy demand and nutrient supply, mitochondria undergo continuous morphological and functional changes [147-149]. This remarkable plasticity allows mitochondria to adapt to abrupt changes in cellular energy demands and reduce cellular stress [150, 151]. Tissue-specific stem cells are capable of both self-renewal and differentiation into at least one mature cell type, which is highly regulated by their interaction with the microenvironment. Mitochondria are key players in enabling stem cells to respond and adapt to environmental stimuli, ensuring cellular homeostasis [152]. Upon osteogenic induction, energy production shifts from glycolysis to oxidative phosphorylation in BM-MSCs or SSCs [153, 154]. Moreover, mitochondrial oxidative phosphorylation is necessary for osteogenic commitment and serves as a prominent energy-driving force for osteogenesis [153]. Skeletal myoblasts also switch their metabolic profile from glycolysis to oxidative phosphorylation upon differentiation [155, 156]. This metabolic shift requires a dramatic remodeling of the mitochondrial network, involving both mitochondrial clearance and biogenesis [156]. In addition, successful myogenic differentiation requires the dynamin-related protein 1 (DRP1)-dependent mitochondrial fission [157]. Mitochondrial dynamics are essential for maintaining MSC commitment; mitochondrial morphology and its regulatory processes of fission and fusion are modulated early on during commitment, leading to alterations in the bioenergetic profile crucial for differentiation [158]. Thus, mitochondrial remodeling correlates with changes in the metabolic program and proliferative state of stem cells, although the specific mitochondrial architecture may not exclusively correlate with specific metabolic or proliferative states [152].

Functional mitochondria are able to adjust and respond to various stressors and metabolic needs [150]. Thus, maintaining mitochondrial homeostasis through the balance of mitochondrial turnover, fission, and fusion is important for tissue function [151, 159]. Conversely, an imbalance in mitochondrial dynamics can disrupt mitochondrial function, leading to tissue dysfunction [160]. Notably, aging affects mitochondrial dynamics; the ratio of Mitofusin 2 (MFN2; a fusion protein) to DRP1 (a fission protein) increases in skeletal muscles derived from old mice compared with young mice [161, 162]. Senescent cells also exhibit decreased expression of DRP1 and mitochondrial fission 1 protein [163]. In addition, although mitochondrial fission activity is specifically induced in SCs after muscle damage, DRP1 activity decreases in old age in both humans and mice [164]. Moreover, reduced mitochondrial fission contributes to the decline in SC regeneration that accompanies aging. The loss of the mitochondrial fission regulator DRP1 in SCs through genetic deletion disrupts the mitochondrial electron transport chain, leading to inefficient oxidative phosphorylation metabolism and mitophagy [164]. This results in increased oxidative stress, reduced proliferation, and functional loss of SCs, ultimately leading to muscle regenerative failure [164].

Furthermore, the peroxisome proliferator-activated receptor gamma coactivator 1 alpha (PGC1α) plays a significant role in mediating mitochondrial homeostasis. PGC1α interacts with multiple transcription factors, including nuclear respiratory factors (NRF1 and NRF2) and mitochondrial transcription factor A (TFAM), which are essential for mitochondrial biogenesis [165, 166]. PGC1α is also directly involved in increasing the expression of proteins related to mitochondrial dynamics such as DRP1 and MFN1 [167]. Thus, PGC1α plays important roles in defending against ROS production and preserving mitochondrial function [168, 169]. Notably, PGC1α expression decreases with aging. PGC1α expression is lower in the SSCs of old mice compared to young mice [170]. PGC1α is downregulated in muscle biopsies from older individuals and in the skeletal muscle of aged mice [171, 172]. The loss of PGC1α with aging is associated with mitochondrial dysfunction and contributes to age-related tissue dysfunction in muscles and bones [170, 173]. Consistently, PGC1α expression rapidly decreases after the induction of skeletal muscle atrophy; conversely, transgenic overexpression of PGC1α protects muscles against atrophy [173]. PGC1α deletion in mice also reduces bone mass but increases the number of osteoclasts and aging-related MAT accumulation as the mice age [170, 174, 175]. In addition, PGC1α expression likely plays a crucial role in the fate decision of MSCs regarding their differentiation towards osteogenesis over adipogenesis [176]. Therefore, decreased PGC1α expression is associated with age-related tissue dysfunction and stem cell senescence. Other proteins related to mitochondrial homeostasis, such as DRP1, may also contribute to mitochondrial dysfunction and tissue degeneration during aging.

The coordinated regulation of mitochondrial remodeling and antioxidant enzymes is required during the differentiation of stem cells [177]. An energy production shift from glycolysis to oxidative phosphorylation increases ROS production, which needs to be balanced by the concurrent upregulation of antioxidant capacity to prevent the accumulation of intracellular ROS [155, 177]. However, aging may hinder the proper upregulation of antioxidant enzymes upon differentiation, thereby increasing the intracellular ROS levels [136]. The resulting ROS accumulation may affect myogenesis and osteogenesis. For example, excessive ROS production contributes to bone remodeling largely by promoting the formation of new osteoclasts and bone resorption [178, 179]. Similarly, high ROS production is associated with decreased osteogenic differentiation induced by cyclic stretching, whereas a ROS scavenger (*e.g.*, *N*-acetylcysteine) reverses this decrease in osteogenesis [136]. Moreover, the level of mitochondrial ROS affects the determination of lineage differentiation [135, 180]. ROS are required for adipocyte differentiation, and mitochondria-targeted antioxidants inhibit adipocyte differentiation and lipid accumulation [181]. Studies have shown that decreased BMD is associated with higher oxidative stress index values, suggesting the involvement of ROS in bone pathologies, including osteoporosis [179, 182]. Oxidative stress may also be linked to impaired skeletal integrity [180]. In addition, skeletal muscle atrophy is accelerated by increased mitochondrial ROS production [183, 184]. Therefore, mitochondrial dysfunction is an important driver of aging and contributes to age-related degeneration, including skeletal muscle atrophy and osteoporosis, by inducing redox dysregulation [159, 185-187]. Moreover, mitochondrial dysfunction in senescent cells contributes to growth arrest, the development of the SASP, and resistance to cell death [188]. Thus, mitochondrial dysfunction and cell senescence are closely intertwined, and ROS derived from dysfunctional mitochondria may be a key contributor to tissue dysfunction during aging.

#### 4.3.2 Correlation between YAP/TAZ activity and mitochondrial function

YAP/TAZ dysregulation may be correlated with mitochondrial dysfunction, leading to the inactivation of YAP/TAZ. Alternatively, dysregulated YAP/TAZ may induce mitochondrial dysfunction. Both scenarios eventually result in stem cell dysfunction and tissue degeneration [170, 189, 190]. For example, YAP/TAZ can be regulated by PGC1α, which is important for mitochondrial quality control [191]. PGC1α controls the SSC lineage decision by inducing TAZ, which functions simultaneously as both a coactivator of Runx2 and a corepressor of PPARγ, thereby promoting osteogenic differentiation while inhibiting adipogenesis [170]. Moreover, the loss of PGC1α inhibits the induction of TAZ during osteogenesis, promoting adipogenesis and suppressing osteogenesis in SSCs [170]. Thus, the downregulation of PGC1α expression during aging may decrease YAP/TAZ induction during myogenesis and osteogenesis (Fig. 3B)

Conversely, dysregulated YAP/TAZ may induce mitochondrial dysfunction. YAP/TAZ regulates mitochondrial fusion and fission, which are critical for proper mitochondrial remodeling and bioenergetic adaptation [14]. Moreover, inhibition of C2C12 myoblast differentiation induced by the downregulation of YAP is accompanied by a decrease in DRP1 expression [14]. Therefore, tissue degeneration induced by dysfunctional mitochondria may be related to the dysregulation of YAP/TAZ activity. In support of this observation, muscle-specific TAZ knockout in mice decreases mitochondrial mass, respiration, and exercise ability; conversely, TAZ expression stimulates mitochondrial biogenesis through the translational increase of TFAM expression in C2C12 myoblasts [190]. However, TFAM upregulation depends on TEAD-mediated transcriptional increase in Ras homolog enriched in brain like 1 *(Rheb1)*, an activator of the mammalian target of rapamycin complex 1, but not in *Pgc1α* [190]. Another study demonstrated that PGC1α is involved in YAP/TAZ-induced mitochondrial biogenesis; knockdown of TEAD1 downregulates PGC1α and inhibits mitochondrial biogenesis, whereas a mutant YAP that increases binding of YAP to TEAD1 upregulates PGC1α and mitochondrial biogenesis [192]. Thus, YAP/TAZ activation may contribute to mitochondrial function by increasing the expression of *PGC1α* or other genes (*e.g.*, *DRP1* and *RHEB1*) related to the maintenance of mitochondrial homeostasis (Fig. 3A).

The activation of YAP/TAZ contributes to the upregulated expression of antioxidant proteins, protecting cells from oxidative stress caused by a shift in energy production from glycolysis to oxidative phosphorylation during stem cell differentiation [189, 193]. For example, YAP interacts with the FoxO family transcription factor FoxO1 on the promoters of antioxidant genes, such as catalase and manganese superoxide dismutase (*MnSOD*), and increases their transcription, reducing oxidative stress and enhancing cell survival [193]. Under oxidative stress, TAZ interacts with TEAD and forms complexes with the promoter of *NRF2*, leading to the induction of NRF2-mediated upregulation of antioxidant genes (*e.g.*, *NRF2*, *SOD*, and catalase) to enhance antioxidant capacity and mitigate oxidative damage, preventing mitochondrial dysfunction [139, 189]. Thus, YAP/TAZ plays a crucial role in relieving the oxidative stress during myogenesis and osteogenesis. Aging alters the ability to properly activate antioxidant systems, leading to mitochondrial dysfunction [155]. Dysregulated YAP/TAZ may contribute to oxidative damage during stem cell differentiation due to the reduced antioxidant capacity associated with aging.

Furthermore, mitochondria actively participate in mechanotransduction during stem cell differentiation [138, 194]. Mechanical cues and alterations in mitochondrial mechanical properties reshape and reorganize the mitochondrial network [195]. Thus, mitochondrial dynamics may link the mechanical signals induced by ECM and metabolic alterations [137]. Notably, ECM stiffness modulates mitochondrial morphology and function; a stiff ECM at adhesion induces ROS production and alters mitochondrial organization and metabolic programing by activating heat shock factor 1, thereby conferring metabolic adaptation and resistance to oxidative stress [196]. Moreover, emerging evidence suggests the existence of crosstalk between mitochondrial function and ECM-induced mechanosignaling during tissue regeneration [194]. Alterations in ECM properties and mitochondrial homeostasis are relevant in aged tissues; therefore, mitochondrial dysfunction and reduced mechanosensing interplay in tissue dysfunction in aged individuals. Because YAP/TAZ activity is important for mechanotransduction in stem cells, for their proper function (Section 4.2), YAP/TAZ may play an important role in interrelating mitochondrial dysfunction with stem cell dysfunction (Fig. 3).

**Figure 4.**
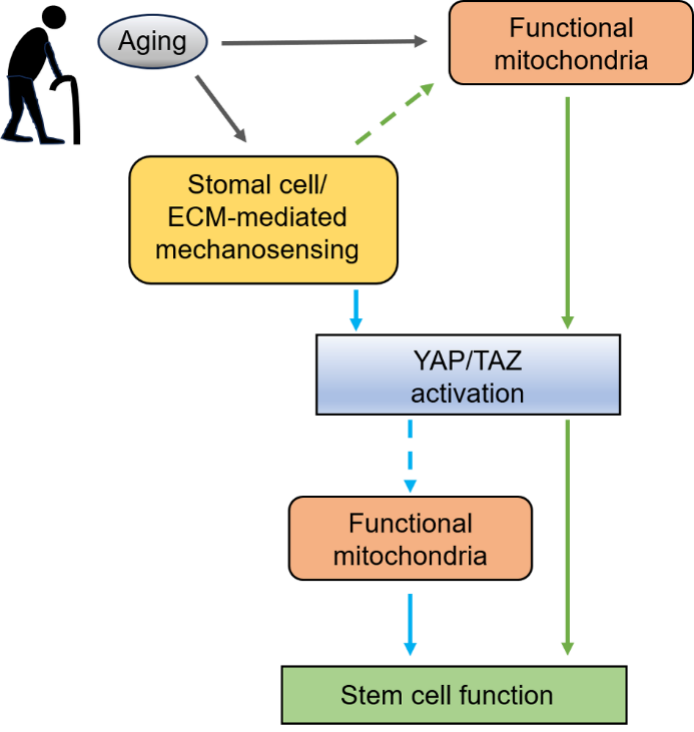
**Effect of aging on the activation of YAP/TAZ and stem cell function**. Stromal cell/ECM-mediated mechanosensing induces the activation of YAP/TAZ, which triggers mechanotransduction in stem cells, promoting their proliferation and differentiation. This mechanosensing can be mediated by functional mitochondria. Alternatively, the activation of YAP/TAZ through mechanosensing is important in maintaining mitochondrial function, which is essential for stem cell function. Aging alters mitochondrial function and stromal cell/ECM-mediated mechanosensing. Aging-related extracellular matrix disorganization may reduce YAP/TAZ activation in stem cells or stromal cells adjacent to stem cells, leading to stem cell dysfunction and senescence (blue). Furthermore, mitochondrial dysfunction during aging may decrease the activity of YAP/TAZ and suppress stem cell function, ultimately contributing to stem cell senescence (green). The resulting accumulation of senescent stem cells can lead to tissue dysfunction, leading to conditions such as skeletal muscle atrophy and osteoporosis.

In summary, the decrease in mitochondrial function and PGC1α expression during aging possibly reduces the YAP/TAZ activity required for preserving stemness, potentially leading to stem cell senescence. Conversely, dysregulation of YAP/TAZ during aging may contribute to the downregulation of genes crucial for mitochondrial homeostasis, including *PGC1α* and *DRP1*, contributing to the loss of stem cell function and subsequent tissue dysfunction. Aging also alters ECM organization, which is essential for transmitting mechanical signals, inducing YAP/TAZ dysregulation in stem cells or stromal cells adjacent to stem cells, ultimately causing a failure in mechanotransduction necessary for stem cell function. Moreover, mitochondria possess mechanosensing ability and can change their shape and function based on ECM stiffness, highlighting the potential role of YAP/TAZ in connecting mechanosensing, mitochondrial function, and stem cell function, which warrants further investigation.

## 5. Conclusions and future direction

The loss of muscle and bone mass progressively increases with aging and largely contributes to disability in the elderly. Age-related skeletal muscle atrophy and bone loss are associated with a reduction in the number of muscle and skeletal stem cells, as well as changes in signaling pathways that activate stem cell proliferation and differentiation into specific lineages during aging due to stem cell dysfunction or senescence. Notably, mechanosensing and mitochondrial function decline with aging, contributing to stem cell senescence and tissue dysfunction.

Compelling evidence suggests that YAP/TAZ play key roles in translating signals derived from the intracellular milieu and extracellular environment into stem cell function. Aging-related ECM disorganization may reduce YAP/TAZ activation in stem cells or stromal cells adjacent to stem cells, leading to stem cell dysfunction and senescence. In addition to mechanosensing impairment, the mitochondrial dysfunction associated with aging (*e.g.*, decrease in PGC1α expression and increase in ROS) decreases YAP/TAZ activity, suppressing stem cell function and promoting stem cell senescence. Alternatively, dysregulation of YAP/TAZ results in the downregulation of PGC1α and other proteins crucial for maintaining mitochondrial function, triggering the production of excessive amount of ROS and senescent phenotypes in stem cells. The resulting accumulation of senescent stem cells causes tissue dysfunction, which manifests as diseases such as skeletal muscle atrophy and osteoporosis (Fig. 4). Thus, YAP/TAZ dysregulation involves reduced mechanotransduction and mitochondrial dysfunction, which are relevant in aged tissues. Restoring the YAP/TAZ regulation disrupted by decreased mechanosensing and mitochondrial dysfunction in stem cells may be crucial for improving age-related dysfunction in muscles and bones. Targeting YAP/TAZ holds great therapeutic potential in reducing age-related muscle and bone dysfunction and enhancing the quality of life of the aging population.

Maintaining stem cell function is also important for preventing other degenerative conditions such as Alzheimer’s diseases. Restoring YAP/TAZ activity has potential for preventing and treating such age-related diseases. Drugs that activate YAP/TAZ are currently in development, but challenges remain regarding their clinical application. One key question is whether YAP/TAZ activation can serve as a general anti-aging strategy, despite the potential risks of developing other age-related diseases such as cancer and tissue fibrosis, given the pro-proliferative and profibrotic roles of YAP/TAZ. Further studies are necessary to elucidate the mechanisms that regulate YAP/TAZ activation during aging and in various pathological condition as well as to identify specific downstream targets to prevent muscle atrophy and osteoporosis. Targeting specific downstream genes or binding partners may be an effective approach for the treatment of musculoskeletal degeneration while minimizing potential complications associated with increased YAP/TAZ activity. Examining the impact of genetic variations on target genes and the combination of interventions, such as exercise, is also important. In addition, further research is needed to clarify the distinct roles of YAP and TAZ in different contexts to improve targeted therapies. Developing better *in vitro* models that accurately mimic human diseases is critical for studying the YAP/TAZ pathway. Factors such as the source of stem cells, cell density, 3D culture methods, and co-culture techniques must be carefully considered to effectively study the YAP/TAZ pathway in human diseases, including muscle atrophy and osteoporosis.
